# NHERF1/EBP50 as a Target for Modulation of MRP Function in HepG2 Cells

**DOI:** 10.3390/ph14030239

**Published:** 2021-03-08

**Authors:** Atsushi Kawase, Miho Hirosoko, Yuka Sugihara, Yunosuke Koyama, Ayaka Fukae, Hiroaki Shimada, Masahiro Iwaki

**Affiliations:** 1 Department of Pharmacy, Faculty of Pharmacy, Kindai University, Osaka 577-8502, Japan; sayama160824@yahoo.co.jp (M.H.); sugihara.yuka@kindai.ac.jp (Y.S.); 1611610010h@kindai.ac.jp (Y.K.); 1611610110k@kindai.ac.jp (A.F.); kindai1350@gmail.com (H.S.); kindai20@gmail.com (M.I.); 2 Pharmaceutical Research and Technology Institute, Kindai University, Osaka 577-8502, Japan; 3 Antiaging Center, Kindai University, Osaka 577-8502, Japan

**Keywords:** transporter, scaffold protein, interaction, peptide

## Abstract

As increased expression and activities of efflux transporters (ETs) often cause drug resistance in cancers, we tried modulating ET activity in cancer cells, using scaffold proteins such as ezrin/radixin/moesin (ERM) proteins, and Na^+^/H^+^ exchanger regulatory factor-1 (NHERF1)/ERM-binding phosphoprotein of 50 kDa (*EBP50*). To see whether EBP50 modulated ET activities in human liver cancer HepG2 cells, we used *EBP50* siRNA and a designed TAT-PDZ1 peptide. The *EBP50* knockdown (*EBP50^KD^*) cells had significantly higher intracellular accumulations of Rho123 and carboxy-dichlorofluorescein (CDF), but not H33342 (i.e., the respective substrates of P-glycoprotein (P-gp), multidrug resistance-associated protein (MRP), and breast cancer resistance protein (BCRP)), compared with control HepG2, suggesting that *EBP50* knockdown in HepG2 cells decreased activity of P-gp and MRP but not BCRP. Treatment with TAT-PDZ1 peptide (>1 pM) resulted in significantly higher CDF accumulation in HepG2 cells, which persisted for ≥180 min after TAT-PDZ1 peptide treatment. These results imply that EBP50 can modulate ET activities. To our knowledge, this is the first report on using a competitive peptide to modulate interactions between MRP and EBP50.

## 1. Introduction

Efflux transporters (ETs) such as P-glycoprotein (P-gp, ABCB1), multidrug resistance-associated protein (MRP, ABCC), and breast cancer resistance protein (BCRP, ABCG2) actively pump anti-cancer drugs out of cancer cells. Increased expression and activities of ETs in cancer cells can thus lead to cancer drug resistance. Use of direct ET inhibitors is not necessarily a promising approach because of low efficacy and/or adverse effects, although inhibition of ETs in cancer cells could help improve the intracellular accumulation of anti-cancer drugs. Therefore, modulation of ET activity in cancer cells could be a novel approach to more effective chemotherapy.

We focused on using a scaffold protein as a target molecule for modulation of ET activity. Scaffold proteins, such as ezrin/radixin/moesin (ERM) proteins, and Na^+^/H^+^ exchanger regulatory factor-1 (NHERF1)/ERM-binding phosphoprotein of 50 kDa (EBP50), exert an anchor function by binding to cytoskeletal proteins, thus participating in plasma membrane localization of ETs. EBP50 is known to link between ETs and ERM proteins [1,2]. For example, *EBP50* knockdown (KD) mice exhibited decreased MRP2 expression on their plasma membranes and lower bile-duct activity [3,4]. We previously demonstrated that knockdown of ERM proteins and ERM-associated proteins decreased ET activity [5,6]. In selecting an ET to modulate in cancer cells for such investigations, an ET with higher expression in tumor cells than normal cells is preferable. As EBP50 is overexpressed in several cancers, such as breast cancer, schwannoma, and hepatocellular carcinoma [7,8,9], it is a candidate target molecule to overcome drug resistance in cancer cells.

Against this background, we evaluated activities of ETs and cytotoxicity by anti-cancer drugs after *EBP50* KD with siRNA. We also determined the effects of a peptide designed to inhibit interactions between MRP2 and EBP50 on MRP activity, because of the peptide’s superior biocompatibility. The present study assesses this novel approach to modulate ET activity in cancer cells.

## 2. Results

### 2.1. EBP50 Knockdown Affected Both mRNA and Protein Levels in HepG2 Cells with siRNA Transfection

**Figure 1:**
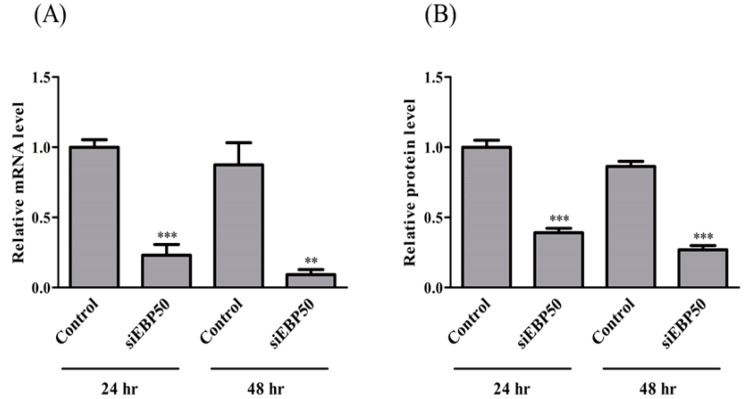
Relative mRNA (**A**) and protein (**B**) levels of EBP50 in control and siEBP50^+^HepG2 cells at 24 and 48 h after siEBP50 treatments. Results are expressed as mean ± SE (*n* = 4). ** *p* < 0.01; *** *p* < 0.001 vs control.

To confirm the efficiency of *EBP50* KD in HepG2 cells by siRNA, mRNA and protein expression levels were determined at 24 h and 48 h after siRNA transfection (Figure 1). Both mRNA and protein levels of EBP50 were significantly decreased at 24 h and 48 h by siRNA KD, with stronger effects at 48 h than at 24 h. We therefore used HepG2 cells 48 h after siEBP50 transfection in these studies.

### 2.2. Activities of P-gp and MRP—But Not BCRP—Increased in HepG2^EBP50KD^ Cells

**Figure 2:**
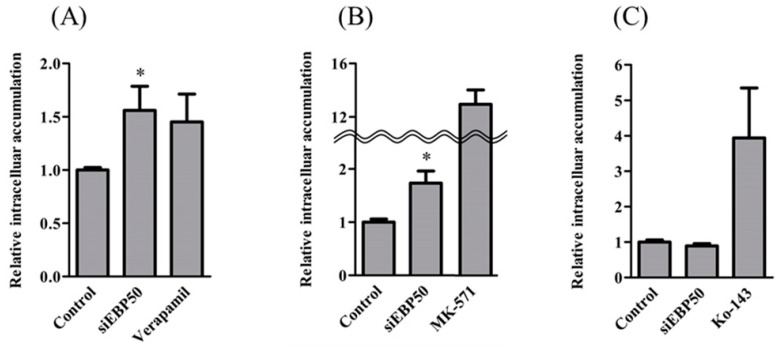
Accumulation of R123 (**A**), carboxy-dichlorofluorescein (CDF) (**B**), and H33342 (**C**) (P-gp, multidrug resistance-associated protein (MRP), and breast cancer resistance protein (BCRP) substrates, respectively) in *EBP50*-knockdown HepG2 cells, 48 h after siEBP50. MRP inhibitor and Ko-143: BCRP inhibitor. Results expressed as mean ± SE (*n* = 3–9). * *p* < 0.05 vs control.

To clarify effects of *EBP50* KD on the transport activity of P-gp, MRP, and BCRP, we determined intracellular fluorescence intensities of transporter substrates in HepG2*^EBP50KD^* cells (Figure 2). The HepG2*^EBP50KD^* cells had significantly greater intracellular accumulation of Rho123 and CDF, but not H33342, than did control HepG2 cells without *EBP50* KD, which indicates that *EBP50* KD in HepG2 cells decreased activities of P-gp and MRP but not BCRP. 

### 2.3. EBP50 KD Led to Decreased Interaction between MRP2 and Radixin in HepG2 Cells

**Figure 3:**
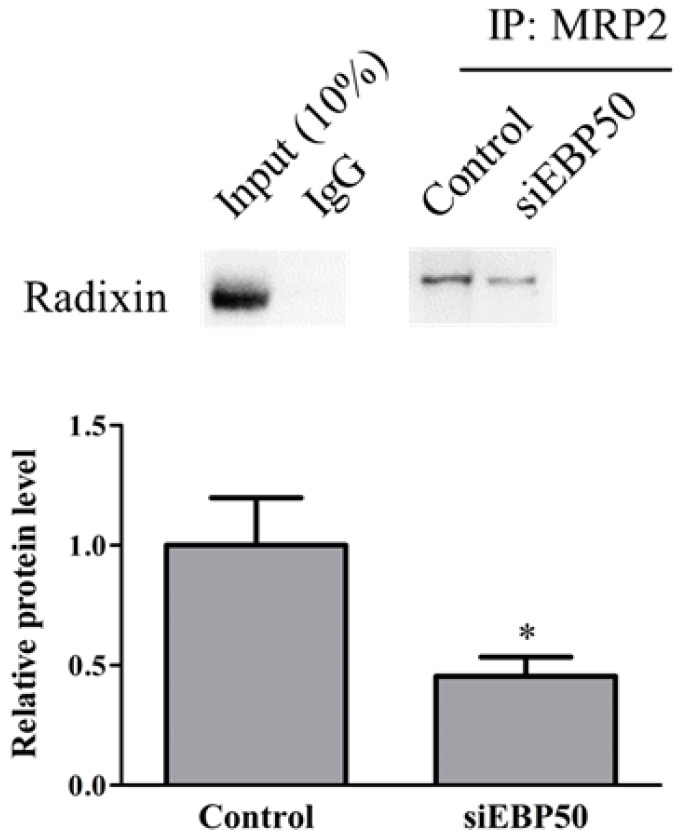
Relative levels of radixin–MRP2 complex formation in lysates of control and siEBP50^+^ cells. IP, immunoprecipitation; IB, immunoblotting. Results expressed as mean ± SE (*n* = 4). * *p* < 0.05 vs control.

To determine whether *EBP50* KD affected interactions between MRP2 and radixin in HepG2 cells, we examined levels of protein–protein complexes by immunoprecipitation assay (Figure 3). The HepG2*^EBP50KD^* cell lysates showed significantly decreased interactions between MRP2 and radixin, compared with controls.

### 2.4. HepG2^EBP50KD^ Cells Showed Increased MTX Efficiency

**Figure 4:**
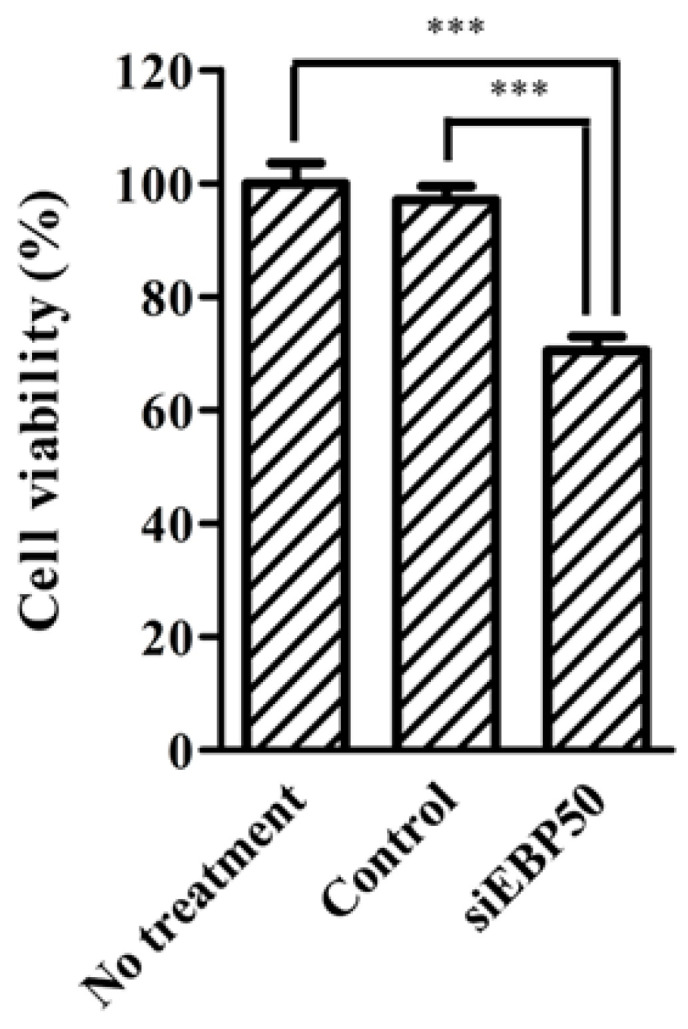
MTT assay shows cell viability after methotrexate (MTX) treatments in *EPB50*-knockdown HepG2 cells. Results expressed as mean ± SE (*n* = 4). *** *p* < 0.001.

Methotrexate (MTX) is an MRP substrate. As MRP activity decreases in HepG2*^EBP50KD^* cells, we considered that MTX efficiency in these cells could increase (Figure 2) and so examined the effect of *EBP50* KD on MTX efficiency in HepG2 cells (Figure 4). MTX efficiency was significantly increased in HepG2*^EBP50KD^* cells compared with untreated cells and control cells.

### 2.5. TAT-PDZ1 Peptide Inhibited MRP Activity in HepG2 Cells

In addition to siRNA KD, we designed a peptide (TAT-PDZ1) to inhibit interactions between MRP and EBP50.

**Figure 5:**
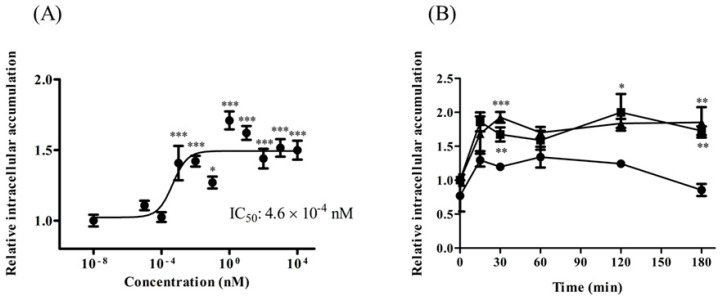
MRP substrate accumulation in HepG2 cells after TAT-PDZ1 peptide treatment at various concentrations (**A**) and times (**B**). Spectrofluorometry shows intracellular CDF accumulation at ●: control, ■: 10 nM, and ▲: 100 nM in (B). Results expressed as mean ± SE (*n* = 4–8). * *p* < 0.05, ** *p* < 0.01, *** *p* < 0.001 vs control.

The TAT-PDZ1 peptide contains TAT peptide (GRKKRRQRRRPQ) and MRP2-PDZ1 core-binding motif (GYGF). TAT-PDZ1 peptide was designed to inhibit EBP50 for MRP2 activity through a competitive mechanism. We found that treatment with TAT-PDZ1 peptide (>1 pM) led to CDF accumulation in HepG2 cells, which was approximately 1.5 times that in untreated controls (Figure 5A). The IC_50_ value was 4.6 × 10^−4^ nM. The effect of TAT-PDZ1 peptide on intracellular CDF accumulation in HepG2 cells persisted for at least 180 min after treating the cells with TAT-PDZ1 peptide (10 nM and 100 nM; Figure 5B). TAT-control peptide alone, without the core-binding motif, had little impact on MRP activity (data not shown), and use of TAT-PDZ1 peptide within the range of concentrations in Figure 5A had little effect on P-gp activity (data not shown).

## 3. Discussion

Our present study showed the potential of EBP50 to modulate the activity of ETs, such as P-gp, MRP, and BCRP, in HepG2 cells. Following treatment with siEBP50 or TAT-PDZ1 peptide, we examined changes in the activity of ETs in HepG2 cells.

The results demonstrated that EBP50 has the potential to modulate ET activity. Several lines of evidence support these findings. First, *EBP50* KD markedly decreased the activities of P-gp and MRP in HepG2 cells. Second, *EBP50* KD decreased interaction between MRP2 and radixin in HepG2 cells, following increased MTX efficiency in HepG2 cells. Finally, the inhibited interaction between MRP2 and EBP50 caused by TAT-PDZ1 peptide decreased MRP activity. 

To our knowledge, this is the first report on using a competitive peptide to modulate interactions between MRP2 and EBP50. We believe that our findings on the effects of TAT-PDZ1 peptide on MRP2 and EBP50 will help us better understand ET regulation in cancer cells and might potentially lead to novel improvements in cancer therapies.

Intracellular accumulations of Rho123 and CDF in HepG2*^EBP50KD^* cells were greater than those in controls (Figure 2). *EBP50* KD, which decreased to approximately 25% of control protein levels (Figure 1B), exhibited significantly decreased activity of P-gp and MRP because the intracellular accumulations of Rho123 and CDF were increased. MRP reportedly interacts with the PDZ1 domain in EBP50 [10,11,12]. However, direct interaction between P-gp and EBP50 is not widely studied, although radixin KD reportedly decreased P-gp activity [13,14]. *EBP50* KD did not affect BCRP activity in HepG2 cells (Figure 2C), suggesting that EBP50 is an effective target to modulate activities of P-gp and MRP, but not BCRP. Decreased P-gp and MRP activity in HepG2 cells could occur via reduced interactions between MRP2 and radixin caused by *EBP50* KD. 

Viability among HepG2*^EBP50KD^* cells after MTX treatment was examined to clarify whether *EBP50* KD affected the anti-cancer efficiency of MTX—an MRP substrate—in these cells (Figure 4). At 1 ng/mL, MTX showed little efficiency in the untreated and control cells (Figure 4). However, HepG2*^EBP50KD^* cells showed greater MTX efficiency at that concentration, probably due to increased intercellular MTX concentrations. Therefore, *EBP50* KD in HepG2 cells might facilitate sufficient MTX efficiency at a lower MTX dose, compared with MTX alone. Conceivably, *EBP50* KD might also improve anti-cancer effects of other MRP substrates, such as cyclophosphamide and cisplatin. Further studies are needed to clarify the effects of *EBP50* KD on the efficiencies of other MRP substrates. 

Use of competitive peptides to modify interactions between proteins is widely applicable to modulate protein functions [15]. The results depicted in Figure 1, Figure 2, Figure 3 and Figure 4 show EBP50’s possible utility as a target to modulate P-gp and MRP activities. We also examined a peptide to inhibit interactions between MRP2 and EBP50 on MRP activity because of the peptide’s higher biocompatibility compared with an siRNA. The TAT-PDZ1 peptide increased accumulation of CDF in HepG2 cells (Figure 5), which suggests that TAT-PDZ1 peptide inhibits MRP activity in HepG2 cells. In this study, the usefulness of EBP50 as a target for modulation of MRP activity in HepG2 cells and the potential of TAT-PDZ1 peptide are shown. However, details of the effects of TAT-PDZ1 peptide on MRP2–EBP50 interactions are unclear, and TAT-PDZ1 peptide’s intercellular localization after being added to HepG2 cells is undetermined. Further studies are needed to clarify the mechanism of how TAT-PDZ1 peptide decreases the activities of MRP in HepG2 cells. 

EBP50 has various roles in cellular functions of cancer cells, in addition to modulating transporter activity. For example, EBP50 affects cellular proliferation and trafficking [16,17], and oncogene activation [18]. Therefore, inhibiting EBP50 may have additional effects on cancer cells, depending on their type.

## 4. Materials and Methods

### 4.1. Chemicals and Reagents

We obtained siRNA for human EBP50 (Silencer Select Validated siRNA, s17921), Stealth RNAi siRNA negative control, Lipofectamine RNAiMAX Transfection Reagent, Opti-MEM I, Fast SYBR Green Master Mix, and BCA protein assay kit from Life Technologies (Carlsbad, CA, USA). We obtained 5(6)-carboxy-2′,7′-dichlorofluorescein diacetate pro-moiety (CDFDA, a precursor of MRP substrate; CDF) and MK571 sodium salt hydrate (an MRP inhibitor) from Sigma-Aldrich; Merck KGaA, Darmstadt, Germany). Methotrexate (MTX, an MRP substrate), Rhodamine 123 (R123, a P-gp substrate), and MS grade porcine pancreatic trypsin were obtained from Wako Pure Chemical Industries (Osaka, Japan). We obtained Hoechst 33342 hydrochloride (H33342, a BCRP substrate) from Cayman Chemical (Ann Arbor, MI, USA). We obtained Ko143 from MedChemExpress (Monmouth Junction, NJ, USA). Dulbecco’s modified Eagle’s medium (DMEM), Sepasol-RNA I Super G, and verapamil were purchased from Nacalai Tesque (Kyoto, Japan). Bond Elut C18 was from Agilent Technologies (Santa Clara, CA, USA). ReverTra Ace was from Toyobo Life Science (Osaka, Japan). Protein G Sepharose 4 Fast Flow was from GE Healthcare (Princeton, NJ, USA). CellTiter-Glo Luminescent Cell Viability assay was obtained from Promega (Madison, WI, USA). Mouse monoclonal anti-MRP2 antibody (M2III-6), rabbit monoclonal anti-radixin antibody (EP1862Y), and mouse anti-IgG antibody were commercially obtained from Abcam (Cambridge, UK). Oligonucleotide primers were from Eurofins (Luxembourg, Luxembourg). TAT-PDZ1 Peptide was from Hokkaido System Science Co., Ltd. (Hokkaido, Japan). All other chemicals and solvents were of MS grade or the highest commercially available purity.

### 4.2. Cell Culture and siRNA or Peptide Treatment

Human liver cancer HepG2 cells were obtained from the RIKEN Cell Bank (Ibaraki, Japan). Cells were maintained in DMEM supplemented with 10% fetal bovine serum, 100 U/mL penicillin, and 100 μg/mL streptomycin at 37 °C in the presence of 5% CO_2_ and 95% air. HepG2 cells were seeded at 1 × 10^5^ cells/well onto a 24-well plate (Sumitomo Bakelite Co., Ltd., Tokyo, Japan) and were transfected with siRNA (siEBP50, or siNegative, 5 pmol/well) complexed with Lipofectamine RNAiMAX in Opti-MEM. The medium was replaced with fresh DMEM 24 or 48 h after transfection, and the cells were used in downstream experiments. For studies of TAT-PDZ1 peptide (GRKKRRQRRRPQCCLEKGPNGYGFHLHGEKGK), TAT-PDZ1 was added to HepG2 cells at concentrations of 1 × 10^−8^ to 1 × 10^4^ nM, showing little cytotoxicity to HepG2 cells. After 30-min incubation, MRP activity was assayed. For studies of duration of TAT-PDZ1 peptide, TAT-PDZ1 was added to HepG2 cells at concentrations of 10 or 100 nM. After 0–180 min, MRP activity was assayed.

### 4.3. Determination of mRNA Levels by Reverse Transcription-Polymerase Chain Reaction (RT-PCR)

Total RNA was extracted from HepG2 cells 24 and 48 h after siNegative or siEBP50 treatments. The mRNA expression levels were measured by RT-PCR as described previously [19,20]. PCR was performed under the following conditions: 40 cycles of 95 °C for 10 s, 55 °C for 10 s, and 72 °C for 30 s. Oligonucleotide sequences for each mRNA target are shown in Table 1. Data were analyzed using StepOne Software (Life Technologies), using the multiplex comparative method. Target mRNA was normalized to the internal control, glyceraldehyde-3-phosphate dehydrogenase (*GAPDH*).

### 4.4. Determination of Protein Levels by LC-MS/MS-Based Targeted Proteomics

LC-MS/MS-based targeted proteomics were performed according to previously described methods [21,22]. Briefly, 50 μg of cell lysate prepared from HepG2 cells, 24 and 48 h after siNegative or siEBP50 treatments, was reduced, alkylated, and digested by MS grade porcine pancreatic trypsin at 37 °C for 18 h, after which samples were desalted with Bond Elut C18. Eluted samples were dried in a vacuum at 50 °C and resuspended in 100 μL of the initial mobile phase (4.5% acetonitrile with 0.1% formic acid). A 20-μL aliquot of each sample was injected into the liquid chromatography-tandem mass spectrometry (LC-MS/MS) system consisting of an LC system (UltiMate 3000 series, Life Technologies) and a TSQ Endura Triple Quadrupole Mass Spectrometer with electrospray ionization (Life Technologies). For data recording and analysis, Finnigan Xcalibur software ver. 3 (Life Technologies) was used. Results were quantified by monitoring the selected transition reaction (mass/charge ratio, precursor > product) of a surrogate peptide (IVEVNGVCMEGK) for hEBP50 (667.8 > 703.4, 894.4, and 780.3).

### 4.5. Transporter Activity Assays

Transporter activities were assayed according to previously described methods [23]. Briefly, CDFDA (10 μM), R123 (50 μM), and H33342 (10 μM), with or without their respective inhibitors (50 μM MK571, 50 μM verapamil, and 10 μM Ko143), were added 30 min before the HepG2 cells were treated with siRNA for 48 h. After a 1 h-incubation for CDFDA and H33342, or a 2 h-incubation for R123, the cells were washed twice with PBS. The cells’ fluorescence intensity was then measured by a fluorescence microplate reader (SH-9000, Corona Electric Co., Ibaraki, Japan) at Ex/Em wavelengths of 495/530 nm for CDF, 480/530 nm for R123, and 355/460 nm for H33342. 

### 4.6. Immunoprecipitation Analyses

Immunoprecipitation analyses were performed according to previously described methods [5]. Briefly, 50 μg of cell lysate of HepG2 cells, treated with siNegative or siEBP50 for 48 h, was incubated with 40 μL Protein G Sepharose 4 Fast Flow and 8 μL anti-MRP2 antibody overnight. The beads were sedimented at 9000× *g* for 1 min and washed three times with lysis buffer. Finally, 50 μL elution buffer was added and boiled at 95 °C for 5 min. After centrifuging the suspension at 9000× *g* for 5 min, the supernatant was subjected to SDS-PAGE and immunoblotting for radixin.

### 4.7. HepG2 Cell Viability after MTX Treatment

Cell viability was determined by CellTiter-Glo Luminescent Cell Viability assay. After treating HepG2 cells with siNegative or siEBP50 for 48 h, MTX was added to fresh medium at 1 ng/mL [24]. After a 24-h incubation, HepG2 cells were incubated for 10 min with CellTiter-Glo reagent. Their luminescence intensities were measured by luminometer (Junior LB 9509 portable tube luminometer, Berthold technologies, Bad Wildbad, Germany).

### 4.8. Statistical Analysis

Differences in between-group means were analyzed by the Bonferroni test or unpaired Student’s *t*-test. GraphPad Prism 5 (GraphPad Software, La Jolla, CA, USA) was used for statistical analyses. *p* < 0.05 was considered significant.

## 5. Conclusions

EBP50 could be a useful target for modulating ET activities. The approach of using a competitive peptide to modify MRP2–EBP50 interactions suggests a potentially novel drug delivery method.

## Figures and Tables

**Table 1:** Primer sequences used in PCR assays.

Gene	Primer Sequence (5′−3′)	Product Size (bp)
*EBP50*	For CCAGGATCGCATTGTGGAG	201
	Rev CCATTGGTGAAGGGCACAG	
*GAPDH*	For AGGGCTGCTTTTAACTCTGGT	206
	Rev CCCCACTTGATTTTGGAGGG	
